# 716 Autologous Skin Cell Suspension for Dorsal Hand Burn Treatment

**DOI:** 10.1093/jbcr/irae036.260

**Published:** 2024-04-17

**Authors:** Joseph Maestas, Eloise Stanton, Kenzie Cohen, Maxwell B Johnson, Justin Gillenwater

**Affiliations:** Keck School of Medicine of USC, Los Angeles, CA; University of Southern California, Los Angeles, CA; Keck Medicine of USC, Los Angeles, CA; Keck School of Medicine of USC, Los Angeles, CA; University of Southern California, Los Angeles, CA; Keck Medicine of USC, Los Angeles, CA; Keck School of Medicine of USC, Los Angeles, CA; University of Southern California, Los Angeles, CA; Keck Medicine of USC, Los Angeles, CA; Keck School of Medicine of USC, Los Angeles, CA; University of Southern California, Los Angeles, CA; Keck Medicine of USC, Los Angeles, CA; Keck School of Medicine of USC, Los Angeles, CA; University of Southern California, Los Angeles, CA; Keck Medicine of USC, Los Angeles, CA

## Abstract

**Introduction:**

Intermediate and deep partial-thickness dorsal hand burn injuries require timely and appropriate care to preserve both form and function. Split-thickness autografting (STAG) remains the standard of care but requires postoperative immobilization and a large donor site. Autologous skin cell suspensions (ASCS) are prepared from a small piece of autograft and are an alternative to STAG for partial thickness burns. They have minimal donor site morbidity and do not require postoperative immobilization. This study aims to assess the utility of ASCS in the treatment of intermediate and deep partial thickness burns of the dorsal hand.

**Methods:**

This IRB-approved retrospective case series identified patients at a regional burn center with intermediate and deep partial-thickness hand burn injuries who were treated with ASCS alone. Data on injury etiology, demographic information, complications, functional variables, and treatment outcomes were collected.

**Results:**

Ten male right-hand dominant patients with intermediate to deep partial thickness dorsal hand burns were identified, with a mean total body surface area involvement of 12.0±9.3%. On average, patients received ASCS alone for their hand burns on post-burn day 7.3±2.5. All ten patients resumed occupational therapy on postoperative day one. Nine patients had full upper extremity range of motion on day of discharge, which occurred on average 9.1±7.0 days after surgery. Four patients developed hypertrophic scarring without range of motion limitation of the ASCS treated hand. One patient experienced ASCS failure and required autografting for wound closure.

**Conclusions:**

This retrospective case series presents ASCS as an effective wound closure strategy for intermediate to deep partial thickness hand burn injuries. In select patients, ASCS minimizes donor site morbidity and permits immediate upper extremity rehabilitation with excellent functional outcomes..

**Applicability of Research to Practice:**

This research highlights the potential benefits of using ASCS for the treatment of dorsal hand burn injuries and thus suggests a possible change in current burn would management.

